# Positive developmental cascades: Strength development reduces support needs in children

**DOI:** 10.1002/jcv2.70097

**Published:** 2026-01-22

**Authors:** Melody R. Altschuler, Xiaoran Tong, Punit Shah, Edelyn Verona, Julianne M. Bowling, Robert F. Krueger, Roman Kotov, Jordan H. McAllister, Michaela Voit, Sachina Paudel, Scott C. Leon, Shashwat Kala, John S. Lyons

**Affiliations:** ^1^ Center for Innovation in Population Health University of Kentucky Lexington Kentucky USA; ^2^ Department of Health Management and Policy University of Kentucky Lexington Kentucky USA; ^3^ Department of Psychology University of Kentucky Lexington Kentucky USA; ^4^ Kentucky Injury Prevention and Research Center College of Public Health University of Kentucky Lexington Kentucky USA; ^5^ Center for Health, Engagement, and Transformation University of Kentucky Lexington Kentucky USA; ^6^ Department of Biostatistics University of Kentucky Lexington Kentucky USA; ^7^ Department of Psychology University of Bath Bath UK; ^8^ Department of Psychology and Center for Justice Research & Policy University of South Florida Tampa Florida USA; ^9^ Department of Psychology University of Minnesota Minneapolis Minnesota USA; ^10^ Department of Psychiatry Stony Brook University Stony Brook New York USA; ^11^ Department of Psychology Loyola University Chicago Chicago Illinois USA; ^12^ Department of Psychiatry Massachusetts General Hospital Boston Massachusetts USA; ^13^ Department of Psychiatry University of Kentucky Lexington Kentucky USA

**Keywords:** behavioral health, developmental cascades, developmental psychopathology, mental health, sensitive period, strengths, support needs

## Abstract

**Background:**

Strength development in children across a range of psychiatric diagnoses may reduce needs for mental health, social, and functioning support over time. A strength‐based adjunct to child and adolescent mental health may foster the developmental context most helpful for achieving desired outcomes with positive developmental cascading effects.

**Methods:**

We longitudinally examined changes across 5 years in the Child and Adolescent Needs and Strengths Assessment in 2‐ to 18‐year‐old children (*N* = 30,103) from a public mental health system.

**Results:**

First, children who began with a greater number of strengths consistently had fewer support needs, not only at entry but also at one, two, three, four, and five years later. Second, initial strengths appeared to have cumulative positive cascades with reduced support needs over time; each additional strength a child possessed at the beginning of service was associated with a progressively faster decrease in their support needs each subsequent year. Furthermore, developing more strengths during the service period also predicted lower support needs one, two, three, four, and five years later. Finally, the impact of developing strengths over time varied depending on the child's age. Developing more strengths was linked to an increasingly rapid reduction in support needs each year for 2‐ to 5‐year‐olds. In contrast, developing more strengths was linked to a progressively slower reduction in support needs each year for 11‐ to 15‐year‐olds.

**Conclusion:**

We provide empirical support suggesting both the clinical utility of strength‐based behavioral health care and the value of strength development in relation to reduction in support needs as a transdiagnostic clinical dimension. In turn, positive developmental cascading effects during a sensitive period early in development suggest the importance of early intervention. Strength‐based mental health classification and treatment systems can be balanced with a traditional mental health symptom focus to more broadly leverage individuals' abilities for adaptation.

## Key points

**What's known?**

- A strength‐based adjunct to child and adolescent mental health may foster the developmental context most helpful for achieving desired outcomes with positive developmental cascading effects.

**What's new?**

- Positive cascading effects of initial strengths on reduced support needs were evident in 2‐18 year‐old children. An increase in strengths was associated with a faster reduction in support needs during early childhood compared to a slower reduction in support needs during late adolescence.

**What's relevant?**

- Strengths development in relation to support needs is a transdiagnostic clinical dimension with clinical utility in child and adolescent mental health. Practice and policy efforts should focus on strength‐based wellness development for children to facilitate the reduction of support needs and, ultimately, improve education and health care systems to best help children thrive.

## INTRODUCTION

Children referred to mental health services present with complex clinical profiles characterized by dimensional differences in various needs for which external support (e.g., psychotherapy, medication, skills training) may be helpful for achieving individual goals for well‐being (Cicero et al., 2024; Michelini et al., 2019, 2021; Morris et al., 2025). Whereas psychiatric conditions have traditionally been conceptualized as both categorical and deficit‐based, the field is shifting towards more dimensional and strength‐based models to acknowledge how individuals adaptively develop strengths that cut across traditional psychiatric categories. Strength‐based approaches that integrate which assets (e.g., talents, interests, and coping skills) and vulnerabilities (e.g., mental health, social, and functioning support needs) are relevant for the unique goals of children and their families in need of external support may be useful for supporting the development of children as an adjunct to deficit‐based approaches, which would promote a more inclusive field of child and adolescent mental health (Altschuler & Lyons, 2024; Bellato & Seker, 2025; Jacob et al., 2025; Lyons, 2022). The concept of strength‐based approaches stems from both collective scientific theorizing and lived experiences, which recognize the potential of these approaches to support individuals with diverse ways of thinking and acting (Apperly et al., 2024; Blume, 1998; Botha et al., 2024; Dwyer, 2022; Fletcher‐Watson, 2022; Hargitai et al., 2025; Layinka et al., 2024; McLennan et al., 2025; Shah et al., 2025; Shah & Holmes, 2023; Sonuga‐Barke & Thapar, 2021). As such, an overarching aim of the present study is to address whether strength development (i.e., the increase over time in assets that promote well‐being) predicts support needs reduction longitudinally and thus can serve a transdiagnostic clinical dimension with developmental and clinical significance in the field of child and adolescent mental health.

A functional strength‐based assessment approach supports the belief that children and their families in mental health services have unique talents, skills, and life events that can be considered strengths or assets to promote well‐being (e.g., familial, interpersonal, coping skills, educational, vocational, psychological, cultural). This information would be considered in conjunction with assessments of unmet needs that would benefit from external support (e.g., risky behavioral, behavioral/emotional, cultural, life functioning, trauma) (Altschuler & Lyons, 2024; Lyons, 2004, 2009, 2022; Obeid & Lyons, 2011). Indeed, encouraging strengths in children in healthcare settings has been theorized to promote positive child experiences and mitigate the negative effects of adverse child experiences (Frankowski, 2023; Kallapiran et al., 2025). Evidence for the effectiveness of this approach comes from evaluations of strength‐based interventions, which have shown promise for improving outcomes such as hospitalization rates, employment and educational attainment, intrapersonal factors, and mental health (Marques, 2016; Tse et al., 2016). The strength‐based approach takes a person‐centered perspective, rather than centering the psychiatric categories, to determine what is relevant for the unique goals of children and their families in need of mental health support (Altschuler & Lyons, 2024; Jacob et al., 2025). From this framework that considers characteristics in the context of the person's environment, the unique lived experiences of children in context are integrated, thereby shifting focus to what is relevant to goal‐oriented functioning. Despite the promise of strength‐based approaches for child and adolescent mental health care, the nature and timing of strengths development in relation to subsequent support needs in children receiving mental health services has not yet been investigated.

To adequately understand the extent to which strengths promote pathways towards the developmental adaptation of reduced support needs for children, developmental psychopathology theory suggests that it is important to consider the *nature* of change in what is referred to as developmental cascades. That is, there has been longstanding interest in the idea that adaptive and maladaptive functions and behaviors change over time to promote or undermine development (Cicchetti, 2013, 2014; Cicchetti & Curtis, 2007). Developmental cascades describe one potential equifinality pathway for how strengths may lead to reduced support needs, based on theoretical perspectives that emphasize the complexity of individual development across time and context and the interplay of developmental processes (Masten & Cicchetti, 2010). Colloquially known as the “snowball effect”—also termed chain reactions, amplification, spillover, or progressive effects—developmental cascading effects describe when an initially modest change in one domain follows a progressively accelerating trajectory, picking up considerable speed as the degree and extent of change becomes more pronounced. Akin to a snowball rapidly growing as it rolls down a hill, an adaptation is thought to amplify as it improves (i.e., “positive cascade”), whereas a maladaptation is thought to amplify as it worsens (i.e., “negative cascade”). A defining feature of both positive and negative developmental cascades is an escalating change in the outcome over time. Positive developmental cascading effects of strengths on support needs reduction may show a pattern of bonus gains, meaning that more initial strengths and/or increasing strengths over time predict lower support needs in a gradual accelerating pace such that there is a progressively faster decrease in support needs each year based on strengths. In contrast, negative developmental cascading effects of strengths on support needs reduction may show a pattern of diminishing gains, where more initial strengths and/or increasing strengths over time predict lower support needs in a gradual slowing pace such that there is a progressively slower decrease in support needs each year based on strengths.

In addition to assessing whether the *nature* of change in support needs based on strengths development shows evidence of developmental cascades, the *timing* of when these cascading effects may be most evident will inform when it might make the most developmental sense to implement preventative interventions. Indeed, well‐timed and targeted interventions could interrupt negative or promote positive cascades (Masten & Cicchetti, 2010), as sensitive periods are points in development during which there is thought to be heightened sensitivity to specific environmental stimuli, with requisite exposure promoting experience‐expectant developmental processes (Knudsen, 2004). Therefore, longitudinal research is needed to test not only the nature but also the timing of strengths development in relation to subsequent support needs. This investigation would underscore developmental and clinical significance in the field of child and adolescent mental health and inform design of timely and targeted interventions.

### The present study

The goal of the present study was to provide preliminary support for the importance of accumulating strengths as a novel transdiagnostic dimension that fosters the developmental context for achieving desired outcomes of reduced support needs of children in a mental health system. We tested whether strengths development predicted reduced support needs with positive developmental cascading effects, particularly during a sensitive period early in development. We addressed the three following aims: (1) Do children who possess more strengths and who increase their strengths over time show reduced support needs over time? We predicted that children who possess more initial strengths during their first year in mental health care, and those who develop more strengths over time, will experience a decrease in their support needs across the years in treatment. (2) Do strengths exhibit patterns consistent with positive developmental cascades in relation to reduced support needs? Here, we predicted that strengths and support needs will show positive developmental cascading effects over time, with more initial strengths and increasing strengths over time predicting lower support needs at an accelerating pace. (3) Do positive developmental cascades on reduced support needs show evidence of sensitive periods where strengths are most strongly associated with support needs reduction? To this end, we explored whether there was evidence of positive developmental cascades earlier in development versus negative developmental cascades later in development for strengths, suggesting there may be a sensitive period earlier in development where the effect of strengths development on support needs reduction is particularly adaptative.

## METHODS

### Participants and procedure

Children 2 to 18 years‐old served by a public mental health system in a western state of the United States were administered longitudinal assessments of the Child Adolescent Needs and Strengths Assessment (CANS) by certified clinicians (i.e., psychologists, social workers, or psychiatrists from outpatient mental health services) approximately every 6 months for up to 5 years (Lyons, 2009, 2022). The data were originally prepared by the mental health system for program evaluation between 2018 and 2024, and were securely transferred to the data center hosted by the University of Kentucky. The data were provided through a Data Use Agreement between the state and the University of Kentucky. The project was reviewed and approved by the University's Institutional Review Board. This research adheres to the ethical principles of the American Psychological Association and University of Kentucky's ethics guidelines.

From the initial transfer of 165,580 mental health system records on 44,651 children, the present study included 145,063 records of 30,103 children who entered the mental health system at the age of 2 to 18 years‐old and stayed at least 30 days with at least 2 assessment visits (see Table 1 and Supporting Information S1: Appendix S1 for more details). Among the full sample of children in the present study, 2316 (7.7%) entered during early childhood (2‐5 years‐old), 9795 (32.5%) entered during middle childhood (6‐10 years‐old), 12,730 (42.3%) entered during early adolescence (11‐15 years‐old), and 5262 (17.5%) entered during late adolescence (16‐18 years‐old). All children in the present study met criteria for one or more International Classification of Diseases (ICD‐10) mental health diagnostic codes (World Health, 1993, 2004). See Table S1 for counts of primary diagnosis, secondary diagnosis, tertiary diagnosis, and any diagnosis for each ICD‐10 code, which were based on their diagnosis at entry in the mental health system.

**TABLE 1:** Sample demographics.

	All children (*N* = 30,103)	^a^02–05 (*N* = 2316)	06–10 (*N* = 9795)	11–15 (*N* = 12,730)	16–18 (*N* = 5262)	Test statistics (*p*‐value)
Entry age						
Mean (SD)	11.5 (3.85)	4.39 (0.74)	8.18 (1.39)	13.1 (1.41)	16.7 (0.72)	*F*(3,^b^) = 83,353
MD [min‐max]	12 [2, 18]	5 [2, 5]	8 [6, 10]	13 [11, 15]	17 [16, 18]	(*p* < 0.001)
Entry age						
^a^02–05	2316 (7.7%)	2316 (100%)	0 (0%)	0 (0%)	0 (0%)	
06–10	9795 (32.5%)	0 (0%)	9795 (100%)	0 (0%)	0 (0%)	*x* ^2^ (18) = 81.2
11–15	12,730 (42.3%)	0 (0%)	0 (0%)	12,730 (100%)	0 (0%)	(*p* < 0.001)
16–18	5262 (17.5%)	0 (0%)	0 (0%)	0 (0%)	5262 (100%)	
Gender						
Not reported	144 (0.5%)	4 (0.2%)	7 (0.1%)	83 (0.7%)	50 (1.0%)	
Male	14,866 (49.4%)	1339 (57.8%)	5522 (56.4%)	5791 (45.5%)	2214 (42.1%	*x* ^2^ (12) = 613.1
Female	14,863 (49.4%)	972 (42.0%)	4260 (43.5%)	6716 (52.8%)	2915 (55.4%)	(*p* < 0.001)
Trans‐male	182 (0.6%)	0 (0%)	1 (0.0%)	120 (0.9%)	61 (1.2%)	
Trans‐female	48 (0.2%)	1 (0.0%)	5 (0.1%)	20 (0.2%)	22 (0.4%)	
Race						
Not reported	2322 (7.7%)	210 (9.1%)	778 (7.9%)	923 (7.3%)	411 (7.8%)	
White	22,948 (76.2%)	1784 (77.0%)	7641 (78.0%)	9592 (75.3%)	3931 (74.7%)	
Black	553 (1.8%)	36 (1.6%)	158 (1.6%)	256 (2.0%)	103 (2.0%)	*x* ^2^ (18) = 81.2
AI/AN	399 (1.3%)	25 (1.1%)	93 (0.9%)	199 (1.6%)	82 (1.6%)	(*p* < 0.001)
NH/PI	31 (0.1%)	1 (0.0%)	9 (0.1%)	12 (0.1%)	9 (0.2%)	
Asian	144 (0.5%)	8 (0.3%)	35 (0.4%)	67 (0.5%)	34 (0.6%)	
Other	3706 (12.3%)	252 (10.9%)	1081 (11.0%)	1681 (13.2%)		
Ethnicity						
Not reported	16 (0.1%)	0 (0%)	2 (0.0%)	9 (0.1%)	5 (0.1%)	*x* ^2^ (6) = 37.1
Non‐Hispanic	20,369 (67.7%)	1562 (67.4%)	6837 (69.8%)	8499 (66.8%)	3471 (66.0%)	(*p* < 0.001)
Hispanic	9718 (32.3%)	754 (32.6%)	2956 (30.2%)	4222 (33.2%)	1786 (33.9%)	

^a^ Reference category in regression analysis.

^b^ *F*‐test degree of freedom = number of children—number of age groups + 1 = 30,103 − 4 + 1 = 30,100.

### Child and adolescent needs and strengths (CANS)

The CANS is a structured consensus‐based assessment tool used by certified mental health system clinicians to measure child support needs and strengths (Lyons, 2022). The complete CANS manual (including detailed instructions for administering the CANS for clinicians) is available from the corresponding author upon request. See Table S2 for the complete list of indicators and domains of the CANS. The CANS is a component of a semi‐structured comprehensive clinical interview that includes overall questions for administering clinicians to consider for assessment. All clinicians reached reliability on CANS administration annually. Clinicians administering the CANS reach consensus with children and their families on the ratings for each indicator. Questions to consider for the indicators are for both the support needs (e.g., “How is the individual functioning in individual, family, peer, school, and community realms?”) and strengths (e.g., “What individual strengths can be used to support a need”?) modules, as well as more specific questions to consider for each of the CANS indicators within each domain (e.g., “What does the child enjoy doing?”). Clinicians are trained and certified to follow the six key principles of CANS administration: (1) Items were selected because they are each relevant to service/treatment planning; (2) Each item uses a 4‐level rating system that translates into action; (3) Rating should describe the child, not the child in services; (4) Culture and development should be considered prior to establishing the action levels (e.g., if a support needs item is not relevant to the developmental or cultural context of the child, it should be rated as a 0 for no need for action); (5) The ratings are generally agnostic as to etiology; and (6) A 30‐day window is used for ratings in order to ensure assessments stay relevant to the child's present circumstances. Additional details on the CANS and clinical setting for the sample for the present study are described in Vsevolozhskaya et al. (2024).

Each indicator is rated by the clinician on four levels between 0 and 3. The rating anchors for indicators of support needs were: 0 = No evidence, no need for action; 1 = Watchful waiting/prevention, history, suspicion, or contention; 2 = Action, the need is interfering with functioning; and 3 = Immediate/Intensive Action, the need is dangerous or disabling. The rating anchors for indicators of strengths were: 0 = Centerpiece strength, central to planning; 1 = Strength is present, useful in planning; 2 = Identified strength, but must be built or developed to be useful; and 3 = No strength has been identified, efforts are needed to identify and develop potential strength. In the present study, the strength indicators were reverse‐coded so a rating of 0 = lack of strength and 3 = presence of useful strength.

#### Support needs

Support needs were measured as the sum of 65 CANS indicators scores across five support needs domains: (1) Traumatic Experiences (e.g., sexual abuse, physical abuse, emotional abuse, neglect, medical trauma, witness to family violence, witness to community/school violence, natural or manmade disasters, war/terrorism affected, victim/witness to criminal activity, parental criminal behavior, disruption in caregiving/attachment losses, systems involvement), (2) Life Functioning (e.g., family functioning, living situation, social functioning, developmental/intellectual, recreational, medical/physical, sleep, sexual development, activities of daily living, school behavior, school achievement, school attendance, legal issues), (3) Cultural Factors (e.g., language, traditions and rituals, cultural stress), (4) Behavioral/Emotional Needs (e.g., adjustment to trauma, emotional/physical regulation, psychosis, attention/concentration, impulsivity/hyperactivity, depression, anxiety, oppositional behavior, conduct, substance use, attachment difficulties, eating disturbances, behavioral regressions, somatization, anger control, mood disturbance, traumatic grief/separation), and (5) Risky Behaviors (e.g., suicide watch, non‐suicidal self‐injurious behavior, other self‐harm/recklessness, danger to others, sexual aggression, runaway/flight risk, delinquent behavior, decision making/judgment, fire setting, intentional misbehavior, sexually reactive behavior, victimization/exploitation, bullying, cruelty to animals).

#### Strengths

Strengths were measured as the sum of 16 reversely coded CANS indicators in the strengths domain: (1) Family, (2) Interpersonal, (3) Educational Setting, (4) Vocational, (5) Coping Skills, (6) Optimism, (7) Talents/Interests, (8) Spiritual Religious, (9) Community Life, (10) Relationship Permanence, (11) Resilience, (12) Involvement With Care, (13) Use Of Free Time, (14) Peer Influences, (15) Legal Permanency, and (16) Cultural Identity.

### Analytical approach

**FIGURE 1:**
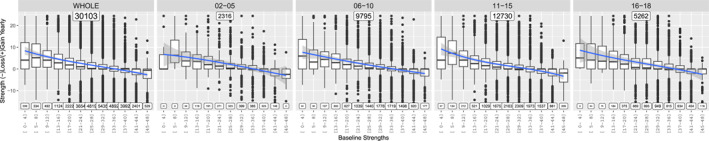
Box‐Wisker plots of baseline strength and rate of change in strength. The *x*‐axis represents baseline strength in 12 equal sized bins from [0–4] to [45–48]. The *y*‐axis represents the rate of losing or gaining strength on a yearly basis past baseline (change in strength divided by length of service). The negative slope fitted across the box‐Wisker revealed common characteristics, such that those who lost strength more rapidly overtime tend to start with higher strength at baseline; conversely, those who gained strength more rapidly overtime tend to start with lower strength at baseline, for the whole sample as well as each age group.

The descriptive statistics for the demographics of the sample are shown in Table 1; the distributions of support needs, strengths, and increase in strengths over time in the full sample and across age groups are shown in Table 2 and Figure 1; the frequency, length, and period of follow‐up assessments in the mental health system are shown Table 3. To examine the impact of child strengths on child support needs, we conducted longitudinal analyses in R (Team, 2013) using linear mixed models with R‐packages lme4 (Bates et al., 2015) and multcomp (Hothorn et al., 2016). We modeled the support needs of each child over time (in years) as the fixed effects of (β1) baseline strengths, (β2) the development of strengths on top of the baseline strengths over time, (β3) the interaction between baseline strength and years in service (i.e., cascade effect of baseline strengths), and (β4) the interaction between the development of strengths past baseline and years in service (i.e., cascade effect of change in strengths), plus the random effect of child‐specific baseline support needs and trajectory of support needs over time. For all analyses, we controlled for child age at entry, gender, race, and ethnicity. For longitudinal analyses examining the effect of strengths trajectories, baseline strengths were controlled for to assess the effect of increasing strength scores on the reduction of support needs above and beyond any variation in baseline strengths. See Supporting Information S1: Appendix S1 for more detail of the mixed model equation and interpretation of terms. We also conducted stratified age analyses by repeating the above modeling within each of the four age groups at entry, while still controlling for variation in age in years within each age group, gender, race, and ethnicity.

**TABLE 2:** Sample distribution of support needs and strengths.

	All children (*N* = 30,103)	^a^02–05 (*N* = 2316)	06–10 (*N* = 9795)	11–15 (*N* = 12,730)	16–18 (*N* = 5262)	Test statistics (*p*‐value)
Baseline support needs						
Mean (SD)	31.8 (18.1)	25.8 (14.5)	29.3 (16.5)	33.6 (18.9)	35.0 (13.3)	*F*(3,^b^) = 245
MD [min, max]	29 [0, 144]	24 [0, 119]	27 [0, 126]	30 [0, 136]	32 [0, 144]	(*p* < 0.001)
Baseline support needs						
None	14 (0.0%)	1 (0.0%)	5 (0.1%)	7 (0.1%)	1 (0.0%)	
1–16	6472 (21.5%)	704 (30.4%)	2402 (24.5%)	2457 (19.3%)	909 (17.3%)	*x* ^2^ (12) = 685
17–32	10,998 (36.5%)	948 (40.9%)	3772 (38.5%)	4500 (35.3%)	1778 (33.8%)	(*p* < 0.001)
33–48	7423 (24.7%)	487 (21.0%)	2400 (24.5%)	3170 (24.9%)	1366 (26.0%)	
49–186	5196 (17.3%)	176 (7.6%)	1216 (12.4%)	2596 (20.4%)	1208 (23.0%)	
Baseline strengths						
Mean (SD)	29.6 (8.60)	28.6 (9.20)	30.4 (8.50)	29.0 (8.50)	29.6 (8.61)	*F*(3,^b^) = 58.8
MD [min, max]	30 [0, 48]	30.0 [0, 48]	31 [0, 48]	29 [0, 48]	30 [0, 48]	(*p* < 0.001)
Baseline strengths						
0–24	8035 (26.7%)	715 (30.9%)	2263 (23.1%)	3658 (28.7%)	1399 (26.6%)	
25–32	10,254 (34.1%)	722 (31.2%)	3218 (32.9)	4472 (35.1%)	1842 (35.0%)	*x* ^2^ (9) = 218
33–40	8884 (29.5%)	708 (30.6%)	3217 (32.8%)	3510 (27.6%)	1449 (27.5%)	(*p* < 0.001)
40–48	2930 (9.7%)	171 (7.4%)	1097 (11.2%)	1090 (8.6%)	572 (10.9%)	
Strength gain^c^						
Mean (SD)	1.05 (5.45)	1.63 (5.74)	1.12 (5.53)	0.873 (5.61)	1.08 (4.73)	*F*(3,^b^) = 13.9
MD [min, max]	0 [−34, 39]	0 [‐34, 33]	0 [‐31, 39]	0 [‐34, 39]	0 [‐33, 35]	(*p* < 0.001)
Strength gain^c^						
Lose 5–34	2532 (8.4%)	159 (6.9%)	789 (8.1%)	1244 (9.8%)	340 (6.5%)	
Lose 1–4	4612 (15.3%)	294 (12.7%)	1493 (15.2%)	2068 (16.2%)	757 (14.4%)	*x* ^2^ (12) = 132
Stable	9930 (33.0%)	780 (33.7%)	3280 (33.5%)	3996 (31.4%)	1874 (35.6%)	(*p* < 0.001)
Gain 1–4	8159 (27.1%)	637 (27.5%)	2622 (26.8%)	3381 (26.6%)	1519 (28.9%)	
Gain 5–39	4870 (16.2%)	446 (19.3%)	1611 (16.4%)	2041 (16.0%)	772 (14.7%)	

^a^ Reference category in regression analysis.

^b^ *F*‐test degree of freedom = number of children−number of age groups + 1 = 30,103 − 4 + 1 = 30,100.

^c^ By the end of follow up.

**TABLE 3:** Sample visits and ICD diagnoses.

	All children (*n* = 30,103)	02–05^a^ (*n* = 2316)	06–10 (*n* = 9795)	11–15 (*n* = 12,730)	16–18 (*n* = 5262)	Test stat (*p*)
Num. of visits						*F*(3,^b^) =
Mean (SD)	4.82 (3.42)	4.95 (3.54)	5.32 (3.77)	4.90 (3.43)	3.64 (2.13)	289.7
MD [min, max]	4.00 [2, 26]	4.00 [2, 23]	4.00 [2, 26]	4.00 [2, 24]	3.00 [2, 17]	(*p* < 0.001)
Num. of visits						
Baseline only	0 (0%)	0 (0%)	0 (0%)	0 (0%)	0 (0%)	
2	8724 (29.0%)	645 (27.8%)	2393 (24.4%)	3601 (28.3%)	2085 (39.6%)	*x* ^2^ (12) =
3	5857 (19.5%)	458 (19.8%)	1783 (18.2%)	2398 (18.8%)	1218 (23.1%)	764.7
4	3957 (13.1%)	294 (12.7%)	1297 (13.2%)	1657 (13.0%)	709 (13.5%)	(*p* < 0.001)
5	2810 (9.3%)	215 (9.3%)	947 (9.7%)	1228 (9.6%)	420 (8.0%)	
6+	8755 (29.1%)	704 (30.4%)	3375 (34.5%)	3846 (30.2%)	830 (15.8%)	
Years follow						*F*(3,^b^) =
Mean (SD)	1.47 (1.21)	1.52 (1.28)	1.65 (1.22)	1.56 (1.22)	0.91 (0.70)	498.8
MD [min, max]	1.04 [0.08, 5.60]	1.04 [0.09, 5.57]	1.24 [0.09, 5.60]	1.18 [0.08, 5.55]	0.69 [0.08, 3.93]	(*p* < 0.001)
Year of entry						
2018	2437 (8.15)	136 (5.9%)	736 (7.5%)	1115 (8.8%)	450 (8.6%)	
2019	6971 (32.1%)	669 (28.9%)	3201 (32.7%)	4128 (32.4%)	1673 (31.8%)	*x* ^2^ (15) =
2020	6040 (20.1%)	488 (21.1%)	1904 (19.4%)	2583 (20.3%)	1065 (20.2%)	94.9
2021	5387 (17.9%)	410 (17.7%)	1749 (17.9%)	2301 (18.1%)	927 (17.6%)	(*p* < 0.001)
2022	4766 (15.8%)	406 (17.5%)	1587 (16.2%)	1917 (15.1%)	856 (16.3%)	
2023	1802 (6.0%)	207 (8.9%)	618 (6.3%)	686 (5.4%)	291 (5.5%)	
ICD diagnosis						
F01‐F09	121 (0.4%)	1 (0.0%)	20 (0.2%)	42 (0.3%)	58 (1.1%)	
F20‐F29	34 (0.1%)	2 (0.1%)	10 (0.1%)	16 (0.1%)	6 (0.1%)	
F30‐F39	77 (0.3%)	0 (0%)	3 (0.0%)	32 (0.3%)	42 (0.8%)	
F40‐F48	5707 (19.0%)	42 (1.8%)	627 (6.4%)	3203 (25.2%)	1835 (34.9%)	
F50‐F59	14,582 (48.4%)	1414 (61.1%)	5096 (52.0%)	5775 (45.4%)	2297 (43.7%)	
F60‐F69	26 (0.1%)	0 (0%)	2 (0.0%)	8 (0.1%)	16 (0.3%)	
F70‐F98	330 (1.1%)	11 (0.5%)	150 (1.5%)	117 (0.9%)	52 (1.0%)	
F99/Z03	9226 (30.6%)	846 (36.5%)	3887 (39.7%)	3537 (27.8%)	956 (18.2%)	

*Note*: F01–F09: Physiological conditions; F20–F29: Schizophrenia spectrum and other psychotic disorders; F30–F39: Mood disorders; F40–F48: Anxiety disorders; F50–F59: Eating and sleep disorders; F60–F69: Personality disorders; F70–F98: Neurodevelopmental disorders; F99–Z03: Other.

^a^ Reference category in regression analysis.

^b^ *F*‐test degree of freedom = num of children − num of age groups + 1 = 30,103 − 4 + 1 = 30,100.

Using the fitted linear mixed model, we projected the support needs across a five year service period (Verbeke, 2000). We limited the projection to a maximum of five years because the availability of follow‐up observations reduced to 1304 individuals (4.3% of the sample) after four years and the assumptions of linear modeling making the projection beyond five years less reliable (Zhao et al., 2001). To improve the reliability of summarized support needs and strengths over time, a median smoother of infinite window was applied to reduce oscillation across assessments and mitigate the impact of longitudinal outlier observations within each child (Justusson, 2006).

Based on reviewer feedback, we also conducted some sensitivity analyses. First, we conducted analyses to examine whether number of visits affects results on strengths, by repeating the above modeling with groups based on number of visits, while still controlling for age in years, gender, race, and ethnicity. We also conducted analyses to examine whether the results are different across children with distinct diagnoses by repeating the above modeling with groups based on ICD‐10 diagnostic categories (Mood, Anxiety, Personality, or Neurodevelopmental Disorders), while still controlling for age in years, gender, race, and ethnicity. Complete results for these sensitivity analyses are reported in Table S3.

## RESULTS

Table 1 describes the demographic characteristics of the sample, which is balanced on gender (49.4% male and 49.4% female), with majority White (76.2%). The sample was more concentrated with 6‐10 year‐olds (*n* = 9,795, 32.5%) and 11‐15 year‐olds (*n* = 12,730, 42.3%) compared to 2‐5 year‐olds (*n* = 2,316, 7.7%) and 16‐18 year‐olds (*n* = 5,262, 17.5%).

Table 2 describes the distribution of support needs and strengths of the sample. The youngest group (2‐5 year‐olds) showed both the fewest strengths and support needs at baseline (*M* = 28.6, SD = 8.6), and the greatest growth during the follow‐up (*M* = 1.63, SD = 5.74). The degree of strengths increased from early childhood (2‐5 years‐old) to middle childhood (6‐10 years‐old) in terms of greater representation in levels with more strengths and less percentage with low/no strengths; however, in early adolescence (11‐15 years‐old) and older adolescence (16‐18 years‐old), the proportions in the higher two levels of strengths went below that of early childhood (2‐6 years‐old). Moreover, children who showed more rapid decreases in strengths overtime tended to start with more strengths at baseline; conversely, children who showed more rapid increases in strengths overtime tended to start with fewer strengths at the baseline. This pattern was evident for the whole sample as well as for each age group (see Figure 1).

**FIGURE 2:**
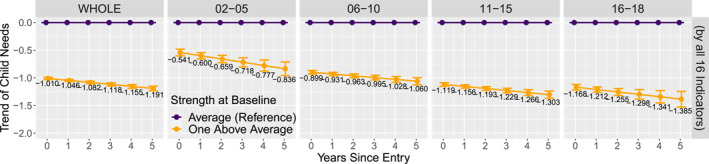
Effect of baseline strengths on support needs. The lines charts (with 95% CI) show the support needs among children who had one more strength than a reference group with average strength at baseline, by the time of entry, and by the time of 1 year, 2 years, 3 years, 4 years, and 5 years since entry, among the whole sample (Panel 1) and per age group (Panel 2–5). The effect line (orange) points downwards in all 5 panels, which suggests the baseline strengths have a bonus effect on lowering support needs overtime, for the entire sample as a whole and for all age groups. If the effect line (orange) were to be parallel to the reference (purple), it would have suggested that baseline strengths have a constant effect of lowering support needs, but without bonus or diminishing effect overtime.

**FIGURE 3:**
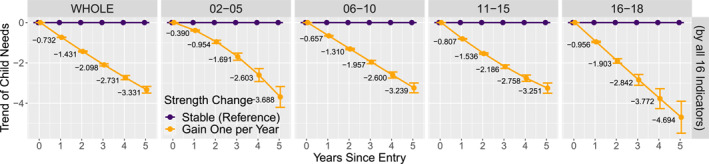
Effect of developing strengths on support needs. The lines charts (with 95% CI) show the support needs among children who gained one strength per year relative to a reference group who did not develop or lose strength, at the time of entry, and by the time of 1 year, 2 years, 3 years, 4 years, and 5 years since entry, among the whole sample (Panel 1) and per age group (Panel 2–5). The effect line (orange) points downwards in all 5 panels, which suggests that developing strength lowered the support needs overtime for both the entire sample as a whole, and for all age groups; the line is a convex in panel 2 (age group 2–5 years‐old), which shows that developing strengths has a bonus effect on lowering the support needs overtime for this age group; on the other hand, the line is a concave in panel 4 (age group 11–15 years‐old), which suggests that developing strengths had a diminishing effect on lowering the support needs overtime for this age group.

Table 3 describes the distribution of study visits and length of stay across age groups. The oldest group (16‐18 years‐old) had both the fewest visits (*M* = 3.64, SD = 2.13) and the shortest follow‐up (*M* = 0.91, SD = 0.70). Results for the effects of strengths on support needs are reported in Table 4. See Table S3 for more detail on the analytical results, including sensitivity analyses for number of visits and diagnostic grouping. Figure 2 demonstrates the effect of baseline strengths on support needs, whereas Figure 3 demonstrates the effect of developing strengths on support needs.

**TABLE 4:** Effects of strengths on support needs.

Strata	Description	Estimate	SE	95% CI	*p*‐value	Cohen's‐d
All ages (*n* = 30,103)	Baseline (constant, β1)	−1.010	0.010	(−1.03, −0.99)	<0.001***	−0.564
	Gained (constant, β2)	−0.749	0.006	(−0.76, −0.74)	<0.001***	−0.696
	Baseline (cascade, β3)	−0.036	0.005	(−0.05, −0.03)	<0.001***	−0.046
	Gained (cascade, β4)	0.017	0.004	(0.01, 0.02)	<0.001***	0.024
Ages 2–5 (*n* = 2316)	Baseline (constant, β1)	−0.541	0.031	(−0.60, −0.48)	<0.001***	−0.370
	Gained (constant, β2)	−0.303	0.018	(−0.34, −0.27)	<0.001***	−0.360
	Baseline (cascade, β3)	−0.059	0.013	(−0.08, −0.03)	<0.001***	−0.096
	Gained (cascade, β4)	−0.087	0.012	(−0.11, −0.06)	<0.001***	−0.148
Ages 6–10 (*n* = 9795)	Baseline (constant, β1)	−0.899	0.017	(−0.93, −0.87)	<0.001***	−0.536
	Gained (constant, β2)	−0.660	0.010	(−0.68, −0.64)	<0.001***	−0.676
	Baseline (cascade, β3)	−0.032	0.007	(−0.05, −0.02)	<0.001***	−0.047
	Gained (cascade, β4)	0.002	0.006	(−0.01, 0.01)	0.691	0.004
Ages 11–15 (*n* = 12,730)	Baseline (constant, β1)	−1.119	0.017	(−1.15, −1.09)	<0.001***	−0.599
	Gained (constant, β2)	−0.847	0.010	(−0.87, −0.83)	<0.001***	−0.749
	Baseline (cascade, β3)	−0.037	0.007	(−0.05, −0.02)	<0.001***	−0.046
	Gained (cascade, β4)	0.039	0.006	(0.03, 0.05)	<0.001***	0.056
Ages 16–18 (*n* = 5262)	Baseline (constant, β1)	−1.168	0.026	(−1.22, −1.12)	<0.001***	−0.621
	Gained (constant, β2)	−0.960	0.018	(−1.00, −0.92)	<0.001***	−0.722
	Baseline (cascade, β3)	−0.043	0.015	(−0.07, −0.01)	0.005**	−0.039
	Gained (cascade, β4)	0.004	0.018	(−0.03, 0.04)	0.817	0.003

*Note*: *n*: number of individuals; Estimate: estimated effect size, a.k.a. the beta; SE: standard error of estimated beta; P: *p*‐values of the test h0: beta/SE = 0; Cohen's *d*: a standardized effect size, as beta/SE/sqrt(N). As an example, according to estimated effect based on the whole sample, the changes in support needs for a child with K strengths at baseline and L strengths gained by the year of T, is expected to be: −1.010 × K + −0.036 × K × T + −0.749 × L + 0.017 × L × T.

### Aim 1: Do children with more strengths, and who develop more strengths over time, experience a reduction in support needs over time?

Negative and statistically significant coefficient estimates for both baseline strengths and change in strengths over time suggest strengths were associated with reduced support needs (Table 4, β1s and β2s). Children who possessed more strengths at baseline showed lower support needs over time one, two, three, four, and five years later in the full sample, for all age groups when stratified by age group at entry, for all number of visit groups when stratified by number of visits, and for all diagnostic groups when stratified by ICD‐10 diagnostic categories. Similarly, children who increased their strengths over time showed fewer support needs over time one, two, three, four, and five years later in the full sample, across all age groups, across all groups based on number of visits, and across all groups based on ICD‐10 diagnostic categories.

### Aim 2: Do strengths show positive developmental cascading effects on reduced support needs?

Effects of baseline strengths on reduced support needs showed a negative and statistically significant interaction between baseline strengths and years (Table 4, β3). In other words, children showed evidence of bonus gains of initial strengths on reduced support needs (i.e., children with more strengths initially had lower support needs each year at a gradual accelerating pace, such that there was a progressively faster decrease in support needs each year based on baseline strengths).

### Aim 3: Do positive developmental cascading effects on reduced support needs show evidence of sensitive periods where strengths are most beneficial?

Effects of strengths development on reduced support needs showed age differences, as evidenced by a significant negative interaction between change in strengths and years for 2‐5 year‐olds, no significant interaction between change in strengths and years for 6–10 or 16‐18 year‐olds, and a significant positive interaction between change in strengths and years for 11‐15 year‐olds (Table 4, β4). In other words, children between 2‐5 years‐old at entry showed evidence of bonus gains of strengths development on reduced support needs (i.e., children between 2‐5 years‐old who increased strengths over time had lower support needs each year at a gradual accelerating pace, such that there was a progressively faster decrease in support needs each year based on change in strengths), children between 6‐10 and 16‐18 years‐old at entry showed no evidence of bonus or diminishing gains of strengths development on reduced support needs (i.e., children between 6‐10 and 16‐18 years‐old who increased strengths over time had lower support needs at the same pace each year), and children between 11‐15 years‐old at entry showed evidence of diminishing gains of strengths development on reduced support needs (i.e., children between 11‐15 years‐old who increased strengths over time had lower support needs at a gradual deaccelerating pace, such that there was a progressively slower decrease in support needs each year based on change in strengths).

## DISCUSSION

Through longitudinal analysis of a large sample of children in a mental health system, we examined the extent to which strengths (i.e., assets that promote well‐being) predicted reduced needs for mental health, social, and functioning support over time across a five‐year period. First, children who started with a greater number of strengths at entry to the mental health system consistently had fewer support needs, not only at entry but also at one, two, three, four, and five years later. Second, each additional strength a child had at the beginning of service was associated with a progressively faster decrease in their support needs each subsequent year. Third, developing more strengths during the service period also predicted lower support needs one, two, three, four, and five years later. Finally, the impact of developing strengths over time revealed a potential sensitive period during which strengths development may be most beneficial for support needs reduction in early childhood. We thereby provide empirical support for the value of strength development in relation to support needs as a novel transdiagnostic clinical dimension that warrants further investigation.

The first aim of the present study was to assess whether children who possess more strengths and who develop their strengths over time show reduced support needs over time. Our findings of baseline strengths and strengths development in association with reduced support needs suggest the balance between strengths and support needs in relation to each child's unique context should become more fully integrated into psychiatric care. Strength‐based approaches in mental health care are starting to gain traction (Ibrahim et al., 2014; Russell et al., 2019; Swartz, 2017; Taylor et al., 2025; Tse et al., 2016; Xie, 2013), but have not yet become widespread enough to be integrated into mental health systems and the field of psychiatry at large. Our results may help inform a shift away from commonly used psychiatric classification systems that focus on pathologizing problems. That is, whereas data‐driven and dimensional approaches to classification often emphasize symptoms of psychopathology (Michelini et al., 2021), these models can be expanded to include measures of dimensional functional support needs and strengths. More specifically, our results suggest classification and treatment systems could benefit from identifying and addressing the unique support needs and strengths of each person in context. The literature already supports a move in this direction, in that neurodiversity approaches highlight how understanding adaptive aspects of individuals' profiles can improve functional wellness (Douglass & Duffy, 2015; Hamilton et al., 2025; Miranda‐Ojeda et al., 2025; Taylor et al., 2023).

The second aim of the present study was to examine whether strengths show patterns of positive developmental cascades on reduced support needs. Our results showing positive developmental cascades of initial strengths on reduced support needs further underscores the critical importance of strengths being harnessed in psychiatric classification and treatment approaches. These findings support the developmental impact of having a “head start,” with more strengths boosting reduction in support needs over developmental time. Aligned with models arguing for the self‐regulation of health (Leventhal et al., 2012) and the neurodiversity literature (Cherewick & Matergia, 2024; Graf‐Kurtulus & Gelo, 2025; Hargitai et al., 2025; Shah & Holmes, 2023), our results suggest that harnessing the unique strengths of each child in mental health services may facilitate support needs reduction with positive developmental cascading effects over time. Our results also align with growing literature that highlights the many neurodivergent strengths that aid in successful adaptation to environmental constraints, such as enhanced prediction of social psychological phenomena (Cherewick & Matergia, 2024; Graf‐Kurtulus & Gelo, 2025; Hargitai et al., 2025; Shah & Holmes, 2023) and creativity (Pennisi et al., 2021; Taylor et al., 2025). Whereas the focus of the present study was on overall strengths development across psychiatric conditions rather than on specific diagnoses or strengths, future work that examines patterns of strengths development across categories of neurodevelopmental and mental health conditions will clarify ongoing debates regarding whether these are distinct versus overlapping categories of neurodiversity (Green, 2023; Knopes, 2025; Miranda‐Ojeda et al., 2025). Results inform an inclusive, integrative framework that more appropriately integrates psychodiagnostic nuances across the full range of neurotypical and neurodivergent forms of ontogenesis rather than the traditional psychopathology approaches that are deficit‐based. The idea is to facilitate “moving toward transdiagnostic dimensional models of neurodiversity and mental health (and away from models of psychopathology)” (Morris et al., 2025).

The third aim of the present study was to explore whether positive developmental cascading effects on reduced support needs show evidence of sensitive periods where strengths may be most beneficial. This was indeed the case for strengths development, in that the increases in strengths over time predicted a faster reduction in support needs in early childhood versus a slower reduction in support needs in late adolescence. We found that children who entered the mental health system in early childhood were the only age group that showed evidence of positive developmental cascading effects of strengths development on reduced support needs. One possible explanation for our age differences findings has implications for developmental considerations of mental health care. That is, those identified and receiving care in early childhood may show positive cascading effects of strengths development on reduced support needs because they are receiving more early intervention services to address their support needs at younger ages. In contrast, older children could represent those who are missed for services until later in life. Given that developmental age is confounded with age of entry into the mental health system in the present study, it is possible that our finding of developmental differences in cascading effects may represent differences in access to services. Alternatively, this could represent the increasing demands of risk problems and other unmet support needs at increasing rates in adolescence (Willoughby et al., 2021), such that focusing on strengths alone may be insufficient. Indeed, the possibility that adolescence is a period of vulnerability for strength development with negative cascading effects on reduced support needs suggests that programs should be tested and developed to consider how to maximize the benefit of strength‐based interventions in this age range by considering the unique challenges for strength development and support needs reduction.

Although we found that middle childhood and late adolescence age groups showed no cascading effects and the early adolescence age group showed negative developmental cascading effects, it is important to note that in the full sample and for each age group, both a) initial strengths (i.e., baseline strengths upon entry in the mental health system) showed positive developmental cascading effects on reduced support needs, and b) strengths development (i.e., changes in strengths over time) were associated with reduced support needs. In other words, we found that strengths were beneficial across age groups, albeit more beneficial in early childhood and less beneficial in early adolescence. These results are suggestive of a potential sensitive period for strengths development and support early efforts to promote strengths development in children while still underscoring the importance of strength identification and building across all age ranges for support needs reduction. Aligned with our results suggesting the positive developmental cascade of initial strengths on reduced support needs is the literature showing that it is becoming more common for children to receive mental health care (Mechanic, 2014) and at younger ages (Horwitz et al., 2014; Kauhanen et al., 2023; Totzeck et al., 2024). Taken together, our results highlight an opportunity to leverage this trend to identify and develop strengths early in childhood while still underscoring the importance of strengths across all ages.

### Limitations and future directions

Results of the present study should be interpreted in the context of its limitations to inform future research and, ultimately, improve practices and policies. Although the large longitudinal sample size of children in a public mental health system is a strength of the study, causality regarding the impact of strengths on support needs, or the type of treatment received, cannot be determined in the present design. Indeed, a lack of information regarding the type of treatment received limits our ability to make conclusions about potential explanations for the age difference findings in cascading effects of strengths development. That is, it is possible that early childhood and adolescent age differences could be indicative of services differences consistent with a later onset of mental health concerns, rather than developmental period differences separate from the receipt of services. A related consideration is that the lack of follow‐up observations in service limited the ability to project beyond five years (Figueroa‐Zúñiga et al., 2013; Swihart et al., 2012; Verkuilen & Smithson, 2012). Future work can examine different types of treatment and test specifically whether affirming models of treatment that balance assets and vulnerabilities can progressively decrease support needs across a wider range of service periods and types when adopting this person‐centered and strength‐based perspective.

Another potential limitation is that strengths and support needs were both measured from the same instrument; thus, shared methodological variance may partially account for the observed pattern. Similarly, clinicians were not blind to the age of children, and it is possible that clinicians systematically rated strengths and support needs differently for younger versus older children. In future studies, it would be useful to control for these possible confounds and to operationalize measures of strengths and support needs with a variety of instruments and informants (e.g., interviews, questionnaires, and observations) and across multiple levels of analysis (e.g., brain, behavior, and cognition) to isolate the effects unique to specific strengths. An experimental approach, where clinicians could be blinded to the age of the child—such as by scoring their strengths and difficulties using vignettes of different aged cases—might also be of interest. Finally, additional variables are likely to contribute to the moderating and likely protective relation between strengths and support needs, and it is likely that specific skills may be particularly impactful for reducing support needs in children. For example, research on compensatory strategies in children suggests that enhanced executive function skills may be a specific asset that is evident among children with adaptive social behavioral presentation despite underlying social cognitive vulnerabilities (Livingston et al., 2019). Future work should build on these lines of research to test the possibility that executive function may moderate the association between strengths development and reduced support needs in children.

### Practice and policy implications

The present results suggest practice and policy implications for improving mental health and education systems. First, results suggest the value in shifting the perspective in assessment and treatment in the field of child and adolescent mental health from one that identifies and defines differences as inherently pathological (e.g., Diagnostic and Statistical Manual of Mental *Disorders*, ICD‐10, Hierarchical Taxonomy of Psycho*pathology*), to a person‐centered perspective that emphasizes the role of supportive cultural and developmental contexts for a balance between support needs and strengths in interaction between a person and their environment (Altschuler & Lyons, 2024; Jacob et al., 2025; Lyons, 2022). To facilitate such a shift, for example, value‐based reimbursement public health system designs (Conrad, 2015; de Silva Etges et al., 2023) could also incentivize strengths identification and development rather than primarily pathology identification and reduction. As another example, the Diagnostic and Statistical Manual of Mental *Disorders* could be renamed to the Diagnostic and Statistical Manual of Mental *Conditions*.

Second, results suggest it would be beneficial for person‐centered, strength‐based questions to be asked and followed up on in treatment to facilitate intentional identification and development of strengths in parallel with identification and reduction of support needs, thereby helping children achieve their individual wellness goals in developmental and cultural context. Some example person‐centered questions for clinicians to consider to identify and develop strengths in clinical practice include: Is the individual able to use their strengths to problems solve and address difficulties or challenges? How does the individual see themselves in the future? What are the things that the individual does particularly well? Taken together, treatment planning in clinical practice could more consistently assess and consider how the unique assets of each individual (e.g., attentional training to increase a strength in coping) can be intentionally developed to achieve their wellness goals (e.g., a reduction in psychotic or anxiety symptoms) rather than focusing as much on the disorders.

Third, given our findings of the particularly beneficial timing of early childhood for strengths development reducing support needs as positive developmental cascades, practice and policy efforts should consider strengths development as a potential evidence‐based intervention of strategic integration of mental health supports in schools (Becker & Domitrovich, 2011; Stephan et al., 2015). The implementation of appropriate structural changes would facilitate integration of this early preventative intervention in education systems for young children with curriculum and resource allocation changes to reflect these shifts.

## CONCLUSIONS

Taken together, our results suggest strengths development in relation to support needs is a transdiagnostic clinical dimension with developmental and clinical significance in the field of child and adolescent mental health, providing preliminary empirical support for the merits of strength‐based approaches in psychiatry as an adjunct to existing deficit‐based models. We found evidence suggesting *positive cascading developmental effects of initial strengths* on reduced support needs across childhood from 2‐ to 18‐years‐old, and of a *sensitive period for the positive cascading developmental effects of strengths development* on reduced support needs in early childhood. Integrating resilience and developmental cascades frameworks (Cicchetti & Curtis, 2007; Masten & Cicchetti, 2010), we provide empirical support for the proposal that strengths identification and building in mental health and education *systems*, particularly during early childhood, may promote positive cascades and counteract negative cascades. Practice and policy efforts should focus on strength‐based wellness development for children to facilitate the reduction of support needs and, ultimately, improve education and health care systems to best help children thrive.

## AUTHOR CONTRIBUTIONS

**Melody R. Altschuler**: Conceptualization; writing—original draft; writing—review and editing; investigation; methodology; project administration. **Xiaoran Tong**: Writing—original draft; writing—review and editing; formal analysis; project administration; methodology; data curation; investigation. **Punit Shah**: Conceptualization; writing—original draft; writing—review and editing; supervision; methodology; investigation. **Edelyn Verona**: Writing—review and editing; conceptualization; supervision; methodology; investigation. **Julianne M. Bowling:** Writing—review and editing; methodology; investigation. **Robert F. Krueger**: Supervision; writing—review and editing; investigation; methodology. **Roman Kotov**: Supervision; writing—review and editing; investigation; methodology. **Jordan H. McAllister**: Data curation; writing—review and editing; investigation; methodology. **Michaela Voit**: Writing—review and editing; data curation; investigation; methodology. **Sachina Paudel**: Writing—review and editing; data curation; investigation; methodology. **Scott C. Leon**: Writing—review and editing; data curation; investigation; methodology. **Shashwat Kala**: Writing—review and editing; investigation; methodology. **John S. Lyons**: Conceptualization; funding acquisition; writing—review and editing; project administration; resources; supervision; data curation; methodology; investigation.

## CONFLICT OF INTEREST STATEMENT

The authors declare no conflicts of interest.

## ETHICAL CONSIDERATIONS

The data were provided through a Data Use Agreement between a state and the University of Kentucky. The project was last reviewed and approved by the University's Institutional Review Board under the Institutional Review Board #55938 on February 18, 2025. Given the usage of secondary data, a waiver of consent was granted by the University of Kentucky's Institutional Review Board under the Institutional Review Board #55938.

## Supporting information

Supporting Information S1

Supporting Information S2

Table S1

Table S2

Table S3

## Data Availability

The data that support the findings of this study are available from the state's public mental health system. Restrictions apply to the availability of these data, which were used under license for this study. Data are available only with the permission of the state's public mental health system.
