# Supplementary material for: Positive developmental cascades: Strength development reduces support needs in children
Source: JCPP Adv. 2026 Jan 22:e70097. Online ahead of print. doi: 10.1002/jcv2.70097 (PMC13338963; doi:10.1002/jcv2.70097)
Supplement: Supplementary file 1 — Supporting Information S1 [file JCV2-9999-e70097-s001.docx]

Positive developmental cascades: Strength development reduces support needs in children

## Supporting Information

## Appendix S1. Detail of Material and Methods

The following sections detail the pre-processing of variables and the linear mixed model employed by the analysis.

***Data Quality Control***

Data quality control (QC) starts with the initial 165,580 records on 44,651 children. The QC first removed records with missing child age and those that predated Jan 1, 2018, and then collapsed pairs of consecutive records with an interval of less than 30 days. This resulted in a sample of 160,250 records for 44,636 children.

Next, the QC fill-up un-triggered CANS ratings with 0s. In the current study, 3 out of 8 domains of CANS were optional (called special modules), and their assessment was triggered by 3 mandatorily assessed items in the remaining 5 core domains. The 3 option domains are Developmental - triggered by Developmental/Intellectual under Life Functioning, Suicide – triggered by Suicide Watch under Risk Behavior, and Runaway – triggered by Runaway/Flight Risk under Risk Behavior. Per-record, if a trigger item was rated 0 or 1 (non-actionable), the corresponding optional domain can be logically filled up with 0s (since they are not assessed). This step did not affect the number of records or children but significantly reduced missing rates in the data.

Some records may still be of low quality due to a higher missing rate even after the 0-fill. The next step removed records with a missing rate greater than 20%. If a child’s record was among those removed, all records for that child were subsequently excluded as we could not establish their baseline. This QC step removed 3 records of a single child, retaining 160,247 records of 44,635 children.

The next step restricted the entry age (age at time of the first record) to be between 2 to 18 years, removing all records of children who entered the behavioral health service before age 2 or after age 18, resulting in a sample of 159,541 records for 44,379 children. Lastly, to support a longitudinal analysis, the final step removed those who stayed less than 30 days (a month) or with only with a single assessment visit, resulting in a final sample of 145,063 records for 30,103 children.

## Statistical Model

The longitudinal statistical model was conducted as follows:

s[Yᵢₜ] = Ageᵢ + Genderᵢ + Raceᵢ + Ethnicityᵢ + b₀ᵢ + b₁ᵢ·t + β₁·s[Xᵢ₀] + β₂·s[Xᵢ₀]·t + β₃·s[Xᵢₜ − Xᵢ₀] + β₄·s[Xᵢₜ − Xᵢ₀]·t

where subscripts “i” and “t” indicate a child and a certain year in service, respectively; Y_it_ and X_it_ denote support needs and strengths of the i^th^ child at year t since entry; X_it_ – X_i0_ denotes the development of strengths by said child at year t since entry—that is, the strengths at time t minus the strengths at baseline (i.e., 0 year), where a negative value corresponds to losing strengths overtime—“s” is the median smooth function; Age_i_, Gender_i_, Race_i_, and Ethnicity_i_ are time invariant demographics of the child.

The random effect coefficients b_0i_ and b_1i_ capture the baseline and trajectory of support needs specific to the i^th^ child; fixed effect coefficients β_1_ and β_2_ capture the constant effect of baseline strength and change of strength on support needs throughout the service, while β_3_ and β_4_ capture the bonus/diminishing effect of baseline strength and change of strength by the time of year t. The sum of β_1_ + β_3_*t is the expected change in support needs at year t with each additional strength at baseline; the sum of β_2_ + β_4_*t can be seen as the expected change in support needs at year t associated with each strength built. A positive β_3_ (or β_4_) suggests a positive developmental cascade, that is, higher strengths at baseline (or more strengths increment overtime) predicts lower support needs in a gradual accelerating pace. Conversely, a negative β_3_ (or β_4_) suggests a negative developmental cascade. That is, higher strengths at baseline (or more strengths increment overtime) predicts lower support needs in a graduate deaccelerating pace.
