# Supplementary material for: Positive developmental cascades: Strength development reduces support needs in children
Source: JCPP Adv. 2026 Jan 22:e70097. Online ahead of print. doi: 10.1002/jcv2.70097 (PMC13338963; doi:10.1002/jcv2.70097)
Supplement: Supplementary file 2 — Supporting Information S2 [file JCV2-9999-e70097-s004.docx]

# Supplemental Table Captions

## Table S1. Counts of ICD-10 Diagnostic Codes

The table lists the counts of primary, secondary, and tertiary diagnoses, as well as any diagnosis, for each ICD-10 code.

## Table S2. CANS Domains and Indicators

The table lists indicators and domains of CANS.

## Table S3. Analytical Results

The table lists all analytical results in the form of (1) regression coefficients, and (2) projected effect of strength / developing strengths at baseline and by the end of year 1 to 5. Each row is paired with the analytical configuration (i.e., age strata).
